# Meta-Prediction of Coronary Artery Disease Risk

**DOI:** 10.21203/rs.3.rs-3694374/v1

**Published:** 2023-12-20

**Authors:** Ali Torkamani, Shang-Fu Chen, Sang Eun Lee, Hossein Sadaei, Jun-Bean Park, Ahmed Khattab, Corneliu Henegar, Nathan Wineinger, Evan Muse

**Affiliations:** Scripps Research & Scripps Research Translational Institute; Scripps Research & Scripps Research Translational Institute; Asan Medical Center, University of Ulsan College of Medicine; Scripps Research & Scripps Research Translational Institute; Seoul National University Hospital; Scripps Research & Scripps Research Translational Institute; Scripps Research & Scripps Research Translational Institute; Scripps Research; Scripps Translational Science Institute, The Scripps Research Institute, Scripps Health

**Keywords:** Machine learning, coronary artery disease, omnigenic, polygenic, integrative prediction, prospective prediction, myocardial infarction, heart attack, cardiovascular disease, cardiometabolic disease, meta-prediction

## Abstract

Coronary artery disease (CAD) remains the leading cause of mortality and morbidity worldwide. Recent advances in large-scale genome-wide association studies have highlighted the potential of genetic risk, captured as polygenic risk scores (PRS), in clinical prevention. However, the current clinical utility of PRS models is limited to identifying high-risk populations based on the top percentiles of genetic susceptibility. While some studies have attempted integrative prediction using genetic and non-genetic factors, many of these studies have been cross-sectional and focused solely on risk stratification. Our primary objective in this study was to integrate unmodifiable (age / genetics) and modifiable (clinical / biometric) risk factors into a prospective prediction framework which also produces actionable and personalized risk estimates for the purpose of CAD prevention in a heterogenous adult population.

Thus, we present an integrative, omnigenic, meta-prediction framework that effectively captures CAD risk subgroups, primarily distinguished by degree and nature of genetic risk, with distinct risk reduction profiles predicted from standard clinical interventions. Initial model development considered ~ 2,000 predictive features, including demographic data, lifestyle factors, physical measurements, laboratory tests, medication usage, diagnoses, and genetics. To power our meta-prediction approach, we stratified the UK Biobank into two primary cohorts: 1) a prevalent CAD cohort used to train baseline and prospective predictive models for contributing risk factors and diagnoses, and 2) an incident CAD cohort used to train the final CAD incident risk prediction model. The resultant 10-year incident CAD risk model is composed of 35 derived meta-features from models trained on the prevalent risk cohort, most of which are predicted baseline diagnoses with multiple embedded PRSs. This model achieved an AUC of 0.81 and macro-averaged F1-score of 0.65, outperforming standard clinical scores and prior integrative models. We further demonstrate that individualized risk reduction profiles can be derived from this model, with genetic risk mediating the degree of risk reduction achieved by standard clinical interventions.

## Introduction

Coronary artery disease (CAD) is a highly heritable and leading cause of death worldwide. Genome-wide approaches estimate its heritability at 40%, with a complex underlying genetic architecture^1^. Genetic predisposition to CAD and its contributing risk factors can be summarized as polygenic risk scores (PRSs), which quantify an individual’s underlying genetic predisposition as a weighted sum across risk loci^2,3^. High polygenic risk for CAD has been independently and repeatedly associated with enhanced benefit from lipid-lowering therapy^4–9^. Despite the ability to identify individuals with enhanced benefit from standard prevention strategies, demonstrations that communication of CAD PRS information can enhance adherence to clinical guidelines^10–14^, and calls from thought leaders for the incorporation of CAD PRSs into clinical guidelines^11,12^, there remains significant debate about the utility of PRSs, especially for risk stratification^15–18^.

Typically, studies that demonstrate no benefit of CAD PRSs in risk stratification focus on older populations with existing disease – performing cross-sectional rather than prospective prediction^19–21^. The conception of these studies is sub-optimal, given that the most appropriate scenario for the utility of a risk factor present at birth is the early detection and intervention of future risk, rather than the detection of late, ongoing disease, which is likely better captured by biomarkers downstream of genetics. In fact, studies that have focused on younger cohorts have consistently demonstrated a benefit of CAD PRS in risk stratification^22–26^. Further, due to the heterogeneity of CAD as a disease and the genetic complexity of CAD predisposition, simple linear models are destined to fail to produce generalizable risk estimates and connections to multiple personalized interventions. The use of PRSs either needs to be contextualized to specific populations / interventions where linear models are useful, or, for multi-dimensional clinical decision-making scenarios, PRSs must be incorporated into more complex frameworks that allow for the interaction between multiple contributing genetic and non-genetic risk factors, a pre-requisite for the detection of personalized interventions.

Several strategies to incorporate multiple PRSs and clinical risk factors into unified CAD risk prediction frameworks have been pursued previously. Relatively straightforward attempts to combine single CAD PRSs and traditional risk factors linearly have resulted in marginal but potentially useful improvements in risk stratification^20,27–33^. Layering on some complexity to incorporate CAD risk factor associated variants (i.e. cholesterol and hypertension associations, etc.) into a single CAD-PRS improves prediction further, though any possibility of understanding the degree of contribution from individual risk factors and their interaction with measured risk factors is lost^34–36^. Similarly, combining individual CAD and risk factor PRSs with a wide range of candidate features in a linear manner allows for the identification of key genetic and clinical risk contributors; however, this brute-force strategy again leads to marginal improvements in risk prediction accuracy and fails to identify interactions required for personalized risk reduction recommendations^37–40^. Incorporation of PRSs into ML models introduces the possibility of complex interactions, but while there have been calls for multi-modal ML models with this utility^41–43^, there have been no convincing demonstrations of ML modeling in genetically-informed and actionable risk estimation^44–48^.

Relatively simple approaches, from a genetic risk perspective, have been attempted for the integration of genetic and clinical risk factors in ML models. Some of these attempts have involved complex modeling, but ultimately produced cross-sectional predictions which, while useful and interesting in some contexts, are not useful for prevention^49^. Attempts at prospective prediction have also focused on manually selected CAD-specific PRSs, ignoring the genetic susceptibility of contributing risk factors and diagnoses, and leading to marginal improvements relative to clinical scores^50^. For example, Steinfeldt et al. developed a prospective CAD risk prediction model using 5 manually selected CAD PRSs (and 1 stroke PRS), improving prospective prediction relative to standard clinical scores but lacking any demonstration of personalized prevention. Other attempts have failed to perform feature selection in the context of model performance, again leading to minimal improvement relative to standard clinical scores^51,52^. For example, You et al. developed an ML model for 10-year incident CAD prediction using 645 candidate variables prioritized on the basis of multicollinearity with one another rather than contribution to predictive performance^53^. None of these prior approaches contemplate meta-prediction, which as we will demonstrate, results in the generation of the most important predictive features, which capture hidden unmodifiable risk status not necessarily expressed in biometrics and diagnoses at baseline, and critically, resulting in the identification of at-risk sub-groups with differing risk reduction benefit from standard clinical interventions.

Thus, our omnigenic^54–56^, integrative, meta-prediction framework is differentiated from this prior work by: 1) demonstrating prospective prediction, 2) adopting an omnigenic hypothesis by incorporating numerous PRSs in a unified risk prediction framework, 3) incorporating those PRSs in an ML framework which enables the detection of interactions between risk factors and interventions allowing for the detection of personalized interventions (i.e. CAD PRS interaction with changes in lipid levels leading to greater benefit in high PRS individuals), and 4) utilizing a meta-prediction framework which integrates predictions for numerous contributing risk factors and diagnoses at baseline and in the future to make an ultimate prediction about CAD event risk in the future. Our meta-prediction framework further incorporates unmodifiable risk predictions (based on age, sex, and genetic factors) as well as modifiable risk predictions (which incorporate measured biomarkers) which further aids in the separation of inherited vs acquired sources of risk. Each of these distinguishing factors contributes to the superior predictive performance we observed relative to prior prospective risk prediction work and allows us to derive personalized interventions directly from model predictions.

## Methods

### Study population

**Figure 1:**
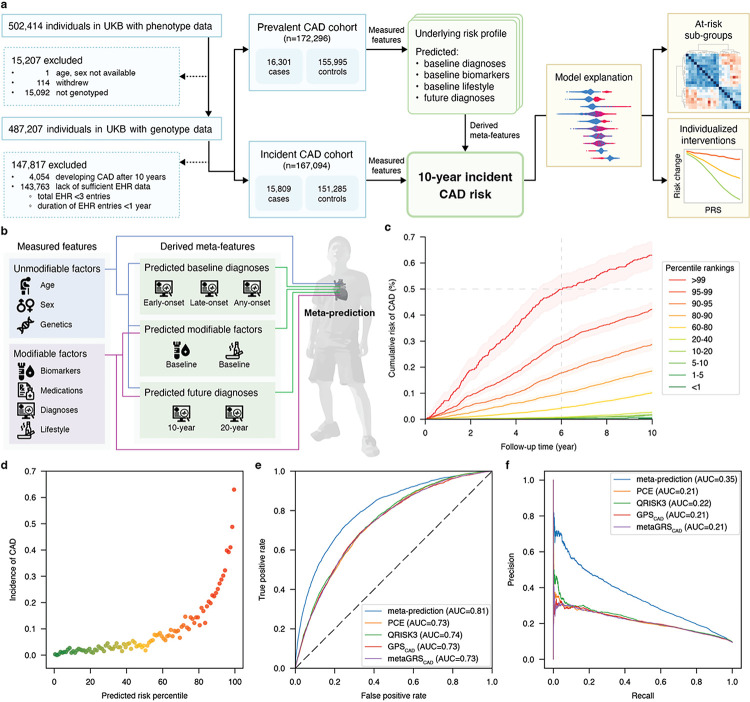
Overview of cohort construction, model development, performance assessment, and inference characterization for 10-year incident coronary artery disease (CAD) risk meta-prediction. **a.** Depiction of primary case cohorts (16,301 prevalent cases with a CAD diagnosis at baseline and 15,809 incident cases developing CAD within 10-years after baseline) derived from the UK Biobank. Controls were filtered to exclude individuals with insufficient EHR data and/or follow-up. **b**. High-level overview of the 10-year CAD risk meta-prediction process, integrating unmodifiable and modifiable risk factors to make predictions about baseline diagnosis, baseline predicted risk factor values, and predicted future diagnoses, which are then all combined to make the final 10-year incident CAD risk meta-prediction. **c**. Cumulative risk curve of CAD (%) development over the 10-year follow-up period stratified by percentile of predicted risk. **d**. Incidence rates of CAD observed across the test cohort, stratified by percentile of predicted risk. **e**. and **f.** Comparative test accuracy (n = 33,419) for our meta-prediction model (AUROC = 0.81; AUPRC = 0.35) versus other standard clinical and research risk scores, including PCE (AUROC = 0.73; AUPRC = 0.21), QRISK3 (AUROC = 0.74; AUPRC = 0.22), GPS_CAD_ (AUROC = 0.73; AUPRC = 0.21) and metaGRS_CAD_ (AUROC = 0.73; AUPRC = 0.21). Abbreviations; **AUC**: Area under curve; **CAD**: coronary artery disease; **EHR**: electric health records; **PCE**: pool cohort equations; **UKB**: UK Biobank.

We defined two primary cohorts (Fig. 1a), aged 40 to 69 at enrollment, from the UK Biobank to power our meta-prediction approach; 1) a prevalent disease cohort of 16,301 individuals with pre-existing CAD at the time of enrollment, and 2) an incident disease risk cohort composed of 15,809 individuals who developed CAD at any time up to 10 years after enrollment. All remaining individuals were considered as possible controls, with exclusion of individuals with insufficient EHR data to confirm lack of CAD.

To arrive at the primary control cohorts, all 470,304 remaining UK Biobank participants were considered as possible controls^57^. 15,207 individuals were excluded due to withdrawal and/or a lack of genotype data. 147,817 individuals were excluded due to lack of sufficient EHR data, defined as those individuals with less than 1 year of follow-up by EHR or less than 3 EHR entries. And 4,054 individuals were excluded due to the development of CAD beyond 10 years of follow-up. After this filtration, 155,995 and 151,285 controls were randomly assigned to the prevalent disease and incident disease cohorts respectively, resulting in a balanced case rate in the two cohorts. The UK Biobank approved the use of the data under application number 41999. This study (IRB-17–7005) was approved by Scripps IRB review board.

### Definition of medical diagnoses and procedures

CAD, the primary outcome, is defined by the appearance of any of the following diagnostic or procedure codes in a subjects EHR, including for heart attack or myocardial infarction from International Classification of Diseases version 10 (ICD-10) codes I21-I24, I25.2 or I46, ICD-9 codes 410–412, 414.2 or 427.5, as well as revascularization and other surgical interventions from Office of Population Censuses and Surveys Classification of Interventions and Procedures version 4 (OPCS-4) codes K40-K46, K47.1, K49, K50, or K75. The date of the event is assigned to the date of the earliest qualifying code. CAD may also be defined by self-report, including responses to the following survey questions: “Vascular/heart problems diagnosed by doctor” response of “1: Heart attack”, “Non-cancer illness code, self-reported” response of “1075: heart attack/myocardial infarction”, “Operation code” response of “1070: coronary angioplasty (ptca) +/− stent”, “1095: coronary artery bypass grafts (cabg)” or “1523: triple heart bypass”. Age of self-report was used to determine the date of the event. Age of CAD was additionally used to further sub-divide the prevalent CAD cohort into an early onset (< 55 years old) and late onset (≥ 55 years old) cohort, allowing for the development of baseline risk models for CAD at any time, as well as early onset vs late onset CAD.

The definition of all 31 other contributing medical diagnoses and procedures used in this work are provided in **Supplementary Table 1**. These additional contributing diagnoses are used in three different ways: 1) they are used as outcomes to generate baseline prevalent risk models and predictions, 2) they are used to make prospective risk models and predictions, including those for secondary CAD diagnoses occurring in the prevalent CAD cohort, and 3) the actual occurrence and duration of diagnoses prior to baseline were used directly as predictive features. To generate baseline and incident risk prediction models for these diagnoses, the prevalent CAD cohort was also stratified into case-control cohorts by these additional diagnoses. Case-control numbers for each of these contributing diagnoses can be found in **Supplementary Table 2**. Controls for these cohorts were filtered to remove individuals with no new EHR entries after the first visit or who died during follow-up due to other reasons.

### Modifiable predictive feature prioritization and pre-processing

The initial set of predictive features, not including genetic features, considered for model development was based on established primary prevention risk models^58–60^ and input from experienced physicians and domain experts on the study team (**Supplementary Table 3**). 36 socio-demographic, 37 family history, 24 self-reported medication use, and 31 lifestyle features were included, collected through a questionnaire administered at the time of recruitment. 22 physical measurements and 63 laboratory tests also collected at the time of recruitment were included. Categorical features were first encoded using the CatBoost Encoding^61^. Repeated measures were aggregated by taking the mean on continuous measures and mode on ordinal measures. Data fields either missing in up to 20% of entries or recorded as “Do not know” or “Prefer not to answer” were subject to imputation using the multiple imputation by chained equations (MICE), incorporating all available variables including those with complete data, using miceRanger package with 5 iterations^62^. 12 features known to be important to CAD risk with greater than 20% missingness, for example smoking features, were also imputed. The preservation of the distribution of variable fields before and after imputation is demonstrated in **Extended Data Fig. 1**. A total of 21 medication variables were created by matching drug name keywords found in both verbal-interview data (**Supplementary Table 4**) and general practitioner (GP) primary care records (**Supplementary Table 5**). Medication variables were excluded from the imputation process.

In addition to the previously described EHR based phenotypes, another 28 synthetic features based on combinations of individual fields were defined and determined post-imputation (**Supplementary Table 3**). We also calculated 2 conventional CAD risk scores; the Pooled Cohort Equations (PCEs) and QResearch Cardiovascular disease Risk Algorithm (QRISK) using modified open-source modules: PCE (https://github.com/Articulus-Tech/ascvd) and QRISK3 (https://f1000research.com/articles/8-2139).

### Unmodifiable predictive features

PRSs were generated using the array-based genotyping data from the UK Biobank after imputation using the standard weighted sum of allele effect and standardization approach^63^. Genotype imputation was performed using the Haplotype Reference Consortium (HRC) reference panel^64^ and following standard procedures^63^, resulting in 37,995,438 autosomal variants for analysis. We selected 17 PRSs not present in the PGS catalog for cardiometabolic traits based on GWAS summary statistics from the latest large-scale GWASs^63,65–71^. Furthermore, we calculated all 3,664 PRSs defined in the PGS Catalog as of 01 June 2023^72^. To prevent potential overfitting, 2,588 PRSs exclusively derived using UK-Biobank data were excluded. A total of 1,093 standardized scores were retained for ML model training (**Supplementary Table 6**).

Genetic ancestry derived from 5 continental populations was estimated using the ADMIXTURE software using 67,047 ancestry informative markers and the 1000 Genomes reference^73^. Sex and family history was determined by self-report. Age, sex, ancestry, family history, and polygenic risk comprise the set of 1,100 unmodifiable risk factors.

### Feature selection and development of the ML pipelines

Tree-based machine learning models, including XGBoost, LightGBM and CatBoost, were considered for all prediction tasks. Other ML models were considered initially but discarded after exploratory analyses demonstrated consistently superior results from XGBoost, LightGBM and CatBoost. Each cohort was divided into an 80:20 train-test split to assess model performance. The train set was further divided during cross-validation for hyperparameter selection as described below.

For feature selection, to accommodate the large number of initial predictive features, we performed a round of pre-selection applying the Saabas’s approximate algorithm to identify the top 200 important features by the average absolute SHapley Additive exPlanations (SHAP) values^74^. We then used our “zoish” wrapper package, which incorporates the v2 algorithm of the SHAP Tree Explainer from the fasttreeshap package to conduct extensive training trials testing various feature number upper bounds to identify minimal models achieving high predictive accuracy (**Supplementary Table 7**). Hyperparameter tuning was automated using our lohrasb module, which incorporates the Tree-structured Parzen Estimator (TPE) sampler along with the Hyperband pruner provided by optuna.

This structure was used to search for the best hyperparameter configuration within pre-defined bounds (**Supplementary Table 8**). For classification tasks, where possible we included sample weights and stratified sampling approaches to account for case-control class imbalance. For each model, we performed 100 trials to identify the best set of hyperparameters, with their performance evaluated using 10-fold cross-validation within the train set (**Supplementary Table 7**). Models were prioritized by F1 score for classification or R^2^ score for regression. Our wrapper pipelines were implemented within the scikit-learn framework in Python 3.8.3 and are available via Github.

### CAD meta-prediction

Baseline prediction models trained in the prevalent CAD cohort included (Fig. 1b): 1) regression models predicting the baseline values of modifiable risk factors, 2) classification models predicting baseline CAD and CAD component diagnoses as well as contributing diagnoses, and 3) classification models predicting future contributing diagnosis. This body of prediction tasks results in 287 meta-features used in the final incident CAD prediction model.

Meta-features representing baseline predicted values of modifiable risk factors were generated in two ways (**Supplementary Table 9**): 1) by predicting their values using unmodifiable features alone, or 2) by predicting their baseline values using both unmodifiable and modifiable features.

In contrast, meta-features for baseline CAD, CAD component, and contributing diagnosis were generated using only unmodifiable features (in order to avoid reverse causation from modifiable risk factors measured at the first recruitment visit after past diagnoses had occurred). These diagnostic outcomes were modeled in three ways (**Supplementary Table 9**): 1) early onset (< 55 years old), 2) late onset (≥ 55 years old), and 3) onset at any age prior to the first UK Biobank recruitment visit.

To predict future contributing diagnoses, we developed two different but overlapping incident risk models per diagnosis (**Supplementary Table 9**): 1) incident disease onset within 10-years of baseline, and 2) within 20-years of baseline. Since reverse causation is not a concern here, both unmodifiable and modifiable predictive features were included in these models.

Inference in the incident CAD risk cohort was performed using these models trained on the prevalent CAD risk cohort. These predictions were stacked with their measured baseline values to produce a final ensemble model for the prediction of incident CAD risk over 10-years. 10 years risk is the standard risk interval used in clinical decision-making especially around the initiation of lipid lowering therapy.

The full set of baseline features and meta-features were then evaluated using multiple ML algorithms and parameters described previously, with the final XGBoost model selected due to a combination of high accuracy with lower complexity from a feature count and tree depth perspective (**Supplementary Table 10** – final model and parameters is bolded).

### Sub-group identification and therapeutic prioritization

Individual level SHAP model explanation values derived from the final meta-prediction model were used to cluster the prospective case cohort into risk sub-groups. This was achieved with agglomerative hierarchical clustering using Ward’s linkage method and Euclidean distance, resulting in sub-groups with individuals sharing similar overall risk profiles. The number of sub-groups was defined by fixed-height tree cutting of the resultant dendrogram. We then conducted one-way ANOVA across all predictive features and used the resultant η^2^ value to prioritize the predictive features that most strongly distinguish the incident case sub-groups.

To assign control individuals to these incident risk sub-groups, we trained a multi-classification XGBoost model using prospective CAD cases and the same features selected for the meta-prediction. Control individuals were assigned to case sub-groups if they achieved an assignment probability of greater than 0.5. At-risk controls were then defined by elevation of individual risk factors (**Supplementary Table 11**).

The impact of preventive interventions was then calculated by modulating: 1) LDL alongside total cholesterol, 2) HbA1c alongside glucose, and 3) systolic blood pressure (SBP) to standard clinical targets (**Supplementary Table 11**). These modulated clinical risk factors were re-entered into the meta-prediction framework to produce an absolute risk estimate post-intervention. The difference between the individual’s initial and modulated risk prediction was then calculated as their absolute degree of risk reduction.

### Reporting

We followed the MI-CLAIM and TRIPOD checklists for reporting results (**Supplementary Tables 12 and 13**). All statistical analyses were performed in R v4.0.0 or Python v3.8.3. All accuracy metrics are reported on the hold-out test cohort after train:test splitting. Risk sub-groups and their differential risk reduction profiles are reported using the entire incident risk cohort. Comparative analysis to prior published PRS models and standard clinical risk scores was performed by training logistic regression models including PRSs, clinical scores, and/or age and sex as covariates, in the same train:test populations used in our ML model training.

## Results

### Cohort definition

The final study population after quality filtration and case-control assignment included 339,390 participants aged between 40 and 69 who enrolled in the UK Biobank (UKB) from 2006 to 2010. 44.95% were male and 55.05% were female, with 9.46% having a diagnosis of coronary artery disease (CAD). 172,296 of these participants formed the prevalent CAD cohort, with 9.46% of these participants diagnosed with CAD prior to enrollment. For incident CAD prediction, we assembled a cohort of 167,094 participants, 9.46% being diagnosed with CAD after baseline over the 10-year follow-up period (Fig. 1a). Additional demographic and clinical characteristics of the prevalent and incident risk cohorts are available in Table 1. While the size of these cohorts leads to statistically significant differences across all parameters, it can be observed that the absolute magnitude of all baseline values is similar across the prevalent and incident CAD cohorts, with the exception of medication usage. The full set of predictive features and their measures in the prevalent and incident risk cohorts are provided in **Supplementary Table 3** in addition to genetic predictors in **Supplementary Table 6.**

### Meta-feature generation and feature selection

1,465 measured features were used to generate 287 additional meta-features by performing training in the prevalent CAD cohort and inference in the incident CAD cohort (Fig. 1b). **Supplementary Table 14** details the outcomes, whether modifiable or unmodifiable predictive features were used, and the test accuracy of each of these 287 predictive models. By design, models predicting diagnoses prior to baseline tend to include a larger number of PRSs as predictive features, whereas future diagnosis models included multiple PRSs alongside several, relevant, measured risk factors.

After meta-feature generation, a total of 1,752 features were considered for inclusion in the final 10-year incident CAD risk ML model via feature selection. Initial approximate feature selection resulted in 200 top ranked predictive features (**Supplementary Table 15**) of which the 60 most important features were selected for final model inclusion via final model training and SHAP-based feature selection. The final 60 prioritized features included 13 directly measured features, 12 PRSs and 35 meta-features (**Extended Data Fig. 2**). Included among the directly measured predictive features, there are 5 lipid measurements, 2 diabetes-related biomarkers, 3 physical measurements, 1 immune function factor, and 2 smoking factors – all of which have established clinical significance in mediating CAD. The genetic risk features selected for the final model included 5 CAD-PRSs (including one CHD-PRS), 1 lipoprotein A-PRS, 2 type 2 diabetes (T2D)-PRSs, 1 alcohol consumption-PRS, 1 major depressive disorder (MDD)-PRS, 1 coeliac disease-PRS, and 1 lung cancer-PRS. The derived meta-features selected for the final model included 2 predicted modifiable factors, 16 predicted future diagnoses, and 17 predicted baseline diagnoses. These predicted diagnoses included a battery of different cardiovascular events, diabetes, white blood cell leukocyte count and waist-hip ratio. These 35 meta-features effectively resulted in the addition of 97 embedded predictive features, 9 of which were also included as stand-alone features in the final model, while 88 of which were selected for inclusion solely via being embedded in a derived meta-feature. Thus, the final model was composed of 114 unique measured features included either as stand-alone features in the final model or embedded within derived meta-features (**Supplementary Table 16**). Further detail of the predictive features is provided after characterizing the accuracy of our final meta-prediction model.

### Meta-prediction performance for 10-year incident CAD risk

The final meta-prediction model (**Supplementary Table 10** – final model and its performance is bolded) effectively stratified the hold-out test set of the incident CAD cohort (n = 33,419) into distinct risk trajectories. By 6-years of follow-up from baseline, over 50.0% of subjects within the highest percentile bin of predicted risk developed CAD (Fig. 1c). At 10-years of follow-up, cumulative CAD incidence rates spanned from 0.3% in the lowest percentile to 63.0% in the highest (Fig. 1c and 1d). Altogether, the model had an AUROC of 0.81 (95% CI 0.80–0.82) (Fig. 1e) and AUPRC of 0.35 (95% CI 0.33–36) (Fig. 1f). The final model had a true positive rate of 54% (95% CI 52%–56%), true negative rate of 87% (95% CI 87%–88%), false positive rate of 13% (95% CI 12–13%), and false negative rate of 46% (95% CI 44%–48%). This equates to an accuracy of 84% (95% CI 84%–85%), a sensitivity of 54% (95% CI 52%–56%), and a specificity of 87% (95% CI 87%–88%), with an F1 score of 0.40 and a macro-averaged F1 score of 0.65. No individual feature category was able to achieve the performance of the final meta-prediction model, including a model composed solely of meta-features, demonstrating the importance of incorporating directly measured and predicted features for final model performance. Specifically, the 13 measured features achieved an AUROC of 0.69 and AUPRC of 0.19, 12 PRSs with an AUROC of 0.65 and AUPRC of 0.18, and the 35 meta-features with an AUROC of 0.78 and AUPRC of 0.30 (**Extended Data Fig. 3**).

### Comparative Evaluation of Clinical Validity between Meta-Prediction and Contemporary Risk Assessment Tools

The accuracy of our final meta-prediction model significantly and substantially exceeded the accuracy of conventional clinical risk scores and previous polygenic risk score benchmarks. Our model produced improved risk stratification across all percentiles of predicted risk across the 10-year follow-up period with an average 2-fold enrichment of CAD events at 10-years among the top percentile bin (**Extended Data Fig. 4**). The AUROC of our meta-prediction model was on average 10% higher than existing approaches (0.81 vs 0.73–0.74), with AUPRCs 63% higher on average (0.35 vs 0.21–0.22) (Fig. 1e–f). Similarly, a survival-based XGBoost estimator (Debiased BCE algorithm from XGBSE) using the same feature set achieved a similar concordance index (C-index) of 0.80. This C-index exceeded that achieved by applying Cox-PH regression to PCE, QRISK3, GPS_CAD_^27^, and metaGRS_CAD_^34^, in agreement with the performance metrics reported in the original publications for these scores, and again leading to a 10% improvement relative to all prior existing methods (0.80 vs 0.72–0.73) (**Supplementary Table 17**). This improved performance translates to a re-classification index of 0.6 (continuous net re-classification index - NRI), 0.3 (absolute integrated discrimination improvement - IDI), and relative IDI of 5.0 on average (**Supplementary Table 18**).

From a binary classification perspective, our meta-prediction approach led to substantial reclassification improvements relative to existing risk scores. The optimal cutoff point for classification was determined using Youden’s index of the AUROC curve, resulting in an optimal meta-prediction classification cutoff as 0.24. When compared to standard clinical score cutoffs of 7.5% for PCE and 10% for QRISK3, our meta-predictor achieved an NRI of 0.13 over PCE, 0.17 over QRISK3. The optimal cutoff for prior polygenic risk models was similarly determined using Youden’s index, leading to an NRI of 0.22 over GPS_CAD_ and 0.23 over metaGRS_CAD_ (Table 2).

### Robustness of meta-prediction across sub-populations

**Figure 2:**
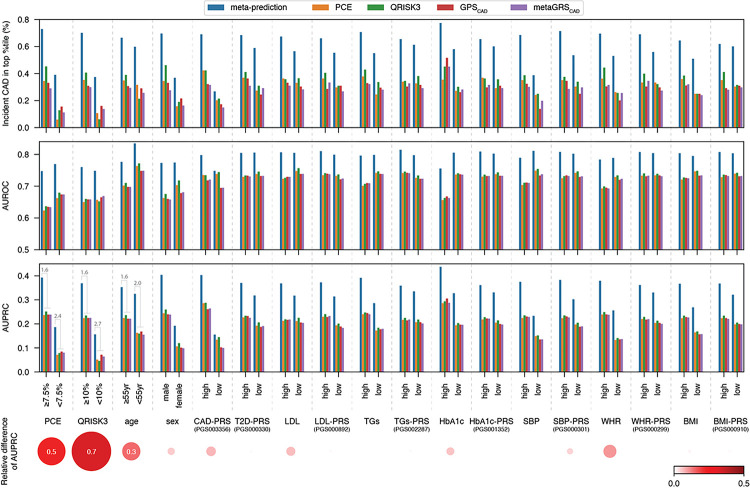
Comparative performance of meta-prediction stratified by standard risk factors. Three-tiered bar charts detailing the meta-prediction’s performance when evaluated across sub-populations stratified by standard clinical risk factors. Model performance is compared with other standard clinical and research risk scores; PCE, QRISK3, GPS_CAD_, and metaGRS_CAD_. The upper bar charts display CAD incidence (%) in the top percentile, the middle bar charts show AUROC values, and the lower bar charts presents AUPRC values. The average fold change in AUPRC of meta-prediction vs other scores is annotated for the three factors showing the greatest advantage of meta-prediction over prior approaches (bottom left bar charts). The bubbles depict the relative difference of these AUPRC-fold change values within each risk factor strata, highlighting those strata where the fold-change in improved performance differs across sub-groups, identifying those risk factors where more than average improvements in performance are achieved for a sub-group. These sub-groups with the greatest gains in performance relative to prior methods include typically low-risk populations (low PCE, low QRISK3, or younger individuals). Abbreviations; **BMI**: body-mass index; **CAD**: coronary artery disease; **PCE**: pool cohort equations; **SBP**: systolic blood pressure; **TGs**: triglycerides; **WHR**: waist-hip ratio.

To ensure model robustness across various CAD risk sub-populations and determine which risk sub-populations may be more effectively captured by our ML model vs prior approaches, we stratified the incident risk cohort by various standard risk factors; PCE, QRISK3, age, sex, CAD-PRS, T2D-PRS, low-density lipoprotein (LDL), LDL-PRS, triglycerides (TGs), TGs-PRS, glycated hemoglobin (HbA1c), HbA1c-PRS, systolic blood pressure (SBP), SBP-PRS, waist-hip ratio (WHR), WHR-PRS, body mass index (BMI), BMI-PRS. Our meta-prediction approach resulted in superior performance across all strata explored, with an average 2-fold improvement in CAD event enrichment in the top percentile of CAD risk, an average 10% improvement in AUROC, and an average 68% improvement in AUPRC per strata, as observed for the overall cohort (Fig. 2 **and Supplementary Table 19)**. Our meta-prediction approach achieved the greatest gains in performance relative to prior methods for individuals with low-PCE (< 7.5%), low-QRISK3 (< 10%), and younger individuals (< 55-year-old), as these groups exhibited enhancements 30–70% improvements in AUC relative to existing approaches (Fig. 2 **and Supplementary Table 20).** These observations are concordant with the known deficiencies in existing risk stratification approaches for capturing at-risk individuals among the typically “low risk.” Although genetic factors are expected to be the primary drivers of risk detection in traditionally low risk individuals, the improved performance among low risk subpopulations included improvements beyond existing PRS-based prediction models, suggesting our improvements are derived from more than just the inclusion of genetic factors.

### Meta-prediction model explanation

To examine the interpretability of our meta-prediction approach, we now describe the measured features that compose the meta-features by examining their importance to those meta-features. Notably, 33 of the 35 meta-features represented predictions of baseline (52%) or future (48%) cardiometabolic or contributing diagnoses, including predictions of abdominal aortic aneurysm, angina, atherosclerotic cardiovascular disease, atrial fibrillation, non-stroke (pre-)cerebral disease, CAD, diabetes, dilated cardiomyopathy, heart failure, myocardial infarction, nonischemic cardiomyopathy, peripheral artery disease, revascularization and stroke risk. The two remaining meta-features were predictions of leukocyte counts and WHR at baseline.

**Figure 3:**
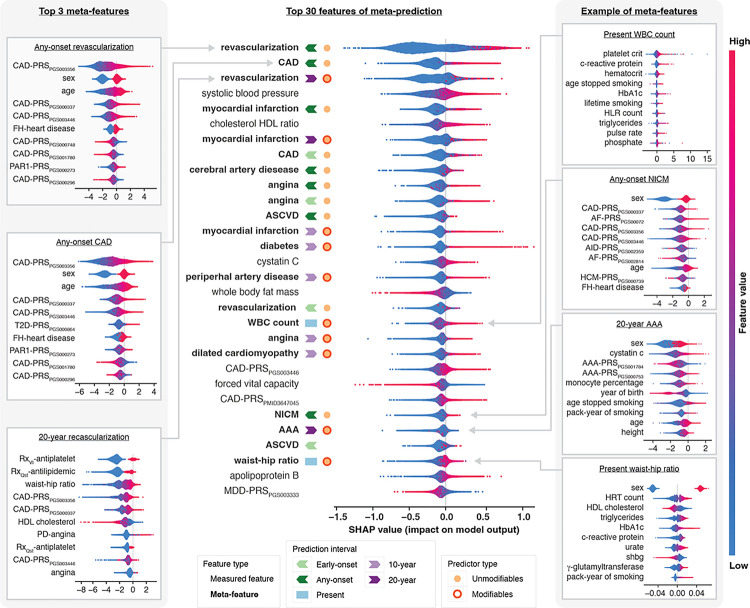
SHAP summary plot of the top 30 features in the meta-prediction framework. This plot displays the top 30 of 60 total features contributing to meta-prediction. The vertical axis orders each feature by its overall importance to risk prediction. Each point represents a participant and is color-coded according to the feature’s direction of contribution to the individuals risk prediction (red increased risk, blue decreased risk). The value associated with each point on the x-axis represents the magnitude of its contribution to the individuals risk prediction. The sub-plots on the left and right provide SHAP plots for selected meta-features, top 3 meta-features on the left, and selected non-CAD future diagnoses on the right. Cerebral artery disease refers to cerebral and pre-cerebral disease other than stroke. Abbreviations; **AAA**: Abdominal aortic aneurysm: **ASCVD**: atherosclerotic cardiovascular disease; **AF**: atrial fibrillation; **AID**: auto immune disease; **FH**: family history; **HCM**: hypertrophic cardiomyopathy; **HLR**: high light scatter reticulocyte; **MDD**: major depressive disorder; **NICM**: nonischemic cardiomyopathy; **PAR1**: protease-activated receptor 1; **PD**: post duration; **Qst**: questionnaire response; **VI**: verbal interview; **WBC**: white blood cell.

Past diagnosis predictions were 41% early-onset, 12% late-onset and 47% any-onset, made using only unmodifiable predictive features. These predictors include several PRSs for cardiovascular conditions, lipid indicators, diabetic elements, immune system features, and a key coagulation activator (protease-activated receptor 1, PAR1), as well as family history. Future diagnosis predictions were 62.5% over 10- and 37.5% over 20-years (Fig. 3 **and Extended Data Fig. 2**). SHAP plots for the top three predictive meta-features are provided on the left side of Fig. 3. The predictive features contributing to these baseline and future risks were largely composed of PRSs, medication use, biomarker measurements, and long-term lifestyle choices. Example SHAP plots for non-CAD or non-CAD component meta-features are provided on the right side of Fig. 3.

The SHAP plots for all meta-features included in the final model are provided in **Extended Data Fig. 5**. Among the 97 features used in these meta-features, we found that age, sex, and 3 CAD-PRSs were the most frequently used predictive factors (**Supplementary Table 16**). 27 meta-feature models contained a non-CAD PRS, including PRSs for contributing diagnoses, related diagnoses, and biomarker levels.

### CAD risk sub-group identification and characterization

**Figure 4:**
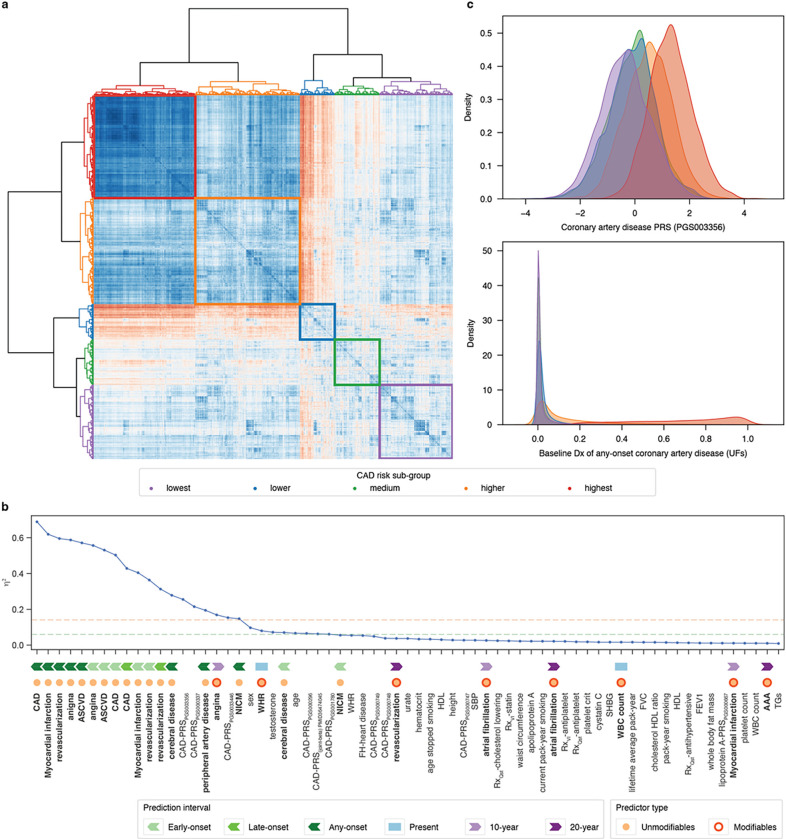
Identification of CAD risk sub-groups and distinguishing features. **a.** A heatmap illustrating the outcome of hierarchical clustering on the SHAP value correlation matrix for all predictors, demarcating five case subgroups in the incident CAD cohort. Each subgroup is assigned a color used in other panels respectively. **b**. A line chart highlighting 57 features with η^2^ values exceeding 0.01 among the five subgroups. Horizontal lines indicate thresholds for moderate (η^2^ ≥ 0.06) and large (η^2^ ≥ 0.14) effects. **c**. Visualization of the distribution of CAD-PRS_PGS003356_ and meta-feature (baseline diagnosis of any-onset CAD predicted by unmodifiable factors) within the 5 subgroups, color-matched to **a**. Abbreviation: **AAA**: Abdominal aortic aneurysm: **ASCVD**: atherosclerotic cardiovascular disease; FH: family history; **FEV1**: forced expiratory volume in 1st second; FVC: forced vital capacity; **NICM**: nonischemic cardiomyopathy; **TGs**: triglycerides **Qst**: questionnaire response; **VI**: verbal interview; **WBC**: white blood cell; **WHR**: waist-hip ratio.

Next, we set out to determine whether our model was able to capture sub-groups of CAD risk that respond differentially to standard clinical risk reducing interventions. As a first step, we identified risk sub-groups in an unbiased manner by clustering individuals by the SHAP values contributing to their overall CAD risk prediction. This approach effectively groups individuals who arrived at their final risk prediction due to similar risk factor profiles. Clustering of the incident cohort CAD cases revealed five distinct subgroups (Fig. 4a). Control individuals were assigned to these sub-groups as described in Methods. A comprehensive summary of the baseline characteristics of each sub-group can be found in Table 3. 67 predictive features were significantly stratified across these subgroups as measured by the ANOVA effect size η^2^ > 0.01. 19 predictive features were differentiated across these sub-groups with a large effect size (η^2^ ≥ 0.14), 9 with moderate effects (0.14 > η^2^ ≥ 0.06), and the remainder with small effects (Fig. 4b). Among the features with large effect size, 15 (79%) were meta-features predicting baseline diagnoses, predicted by unmodifiable risk factors only. 33% of these meta-features were predictions of early-onset events, 13% predicted late-onset events, and 53% predicted events any-time prior to baseline. Notably the three CAD-PRSs (from PGS000337, PGS003446, and PGS003356) and the meta-feature representing baseline prediction of CAD onset using only unmodifiable risk factors showed the greatest degree of stratification across the risk sub-groups. These features differentiated the sub-groups to a greater extent than age or sex as measured by η^2^ (Fig. 4c). Further delineation of sub-groups by further sub-division of the 5 major sub-groups differentiated by standard clinical risk factors including glycated hemoglobin, LDL, and SBP. In other words, measured biometric values defined sub-groups within the major sub-groups primarily defined by their unmodifiable risk profile (**Extended Data Fig. 6).**

### Genetic risk stratification and differential response to clinical interventions

**Figure 5:**
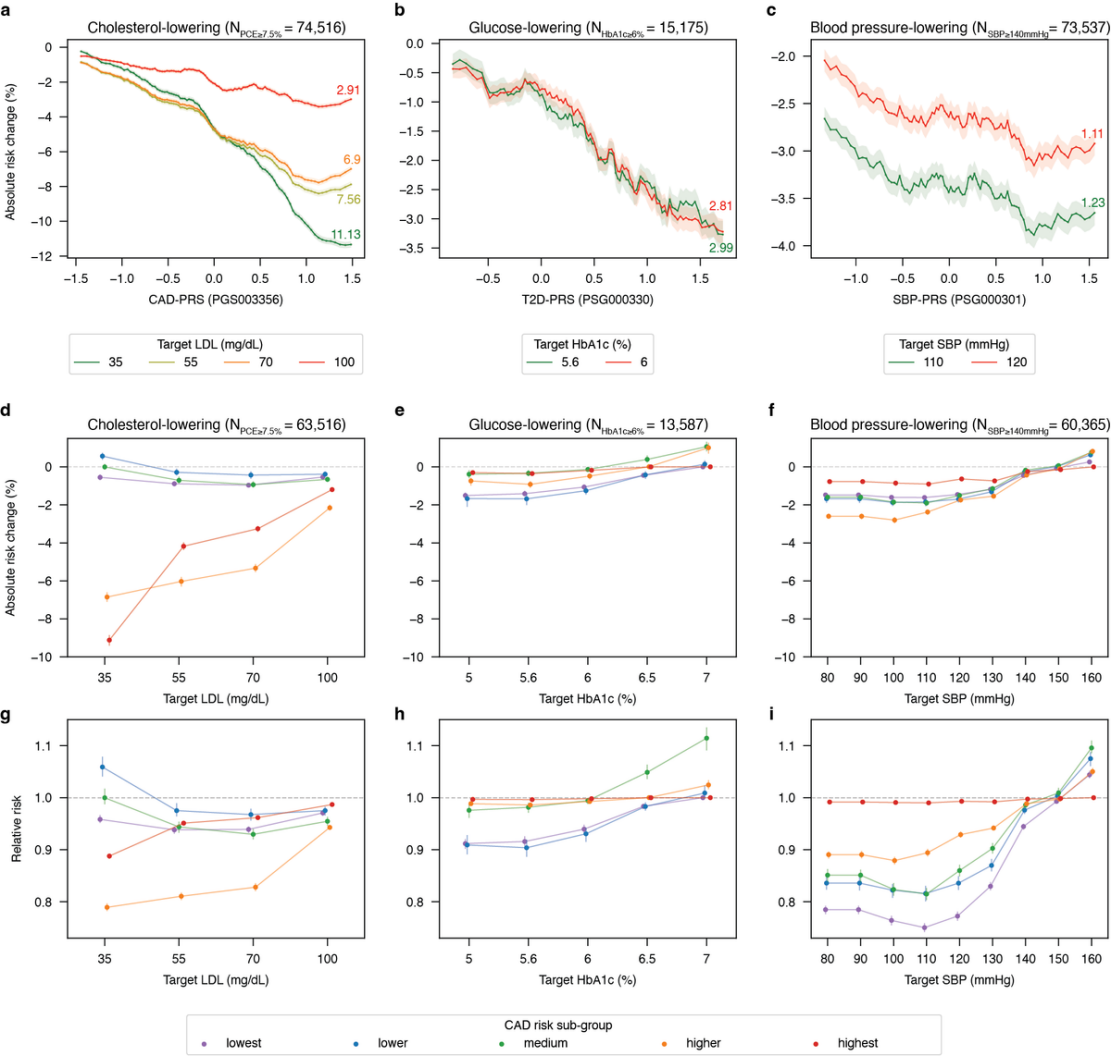
Benefit of clinical interventions by genetic risk and risk sub-groups. Upper panels (**a-c**) relate absolute risk reduction achieved with standard clinical interventions with degree of relevant genetic risk in at-risk individuals. Values are moving averages computed using a rolling window encompassing ±5 percentile bins, with error represented by SEM. Annotated values indicate the maximal benefit achieved per biomarker target: **a.** Absolute risk reduction achieved by LDL-lowering targets of 35, 55, 70, and 100 mg/dL vs standardized CAD-PRS_PGS003356_; **b.** Absolute risk-reduction achieved by HbA1c-lowering targets of 5.6% and 6%/ vs standardized T2D-PRS_PGS000330_; **c.** Absolute risk reduction achieved by SBP-lowering targets of 110 and 120 mmHg by standardized SBP-PRS_PGS002257_. Middle panels present the absolute risk reduction and lower panels present the relative risk change across risk sub-groups. Risk sub-groups are colored according to their assignments in Fig 4. **d.** Absolute risk reduction and **g.** relative risk reduction achieved by LDL lowering targets of 35, 55, 70 and 100 mg/dL. **e.** Absolute risk reduction and **h.** relative risk reduction achieved by HbA1c lowering targets of 5, 5.6, 6, 6.5 and 7%. **f.** Absolute risk reduction and **i.** relative risk reduction achieved by SBP lowering targets of 80, 90, 100, 110, 120, 130, 140, 150, and 160 mmHg. Each data point represents the median, with error bars representing the standard error.

Upon identifying CAD risk sub-groups, we then set out to determine whether individuals within these sub-groups were predicted to respond differentially to standard clinical risk reducing interventions. In particular, we attempted to confirm and demonstrate that the degree of risk reduction achieved through standard preventive interventions was related to underlying genetic risk. Using our trained models, we simulated the influence of risk reducing interventions on the target analyte and performed model inference to determine the change in predicted risk. The clinical interventions, thresholds used to identify at risk individuals, the perturbed biomarker, and their clinical targets described in **Supplementary Table 11**. The degree of absolute CAD risk reduction achieved in the cohort overall by meeting clinical targets for LDL, HbA1c, and SBP by degree of the associated genetic risk is presented in Fig. 5a–c. Similarly, the degree of absolute and relative risk reduction achieved in each CAD sub-group by meeting these targets is presented in Fig. 5d–f and Fig. 5g–i.

Across the population overall, for individuals with a PCE ≥ 7.5%, a widely accepted threshold for the initiation of cholesterol-lowering therapy, the degree of absolute CAD risk reduction increases with increasing CAD genetic risk (Fig. 5a). This is true across all LDL target levels tested. Notably, the degree of risk reduction achieved varied substantially across genetic risk and LDL targets, with the magnitude of risk reduction in high vs low genetic risk individuals ranging from 2.91% at an LDL target of 100 mg/dL to 11.13% at an LDL target of 35 mg/dL. This suggests that low LDL targets are substantially more beneficial for individuals at high genetic risk. Furthermore, when the degree of risk reduction is broken down by risk sub-groups, it can be observed that those individuals in the high CAD genetic risk sub-groups achieved substantially more absolute and relative risk reduction than other risk sub-groups, particularly for the low LDL target of 35 mg/dL (Fig. 5d,g). An absolute risk reduction difference of more than 8% can be achieved in these high genetic risk sub-groups going from an LDL of 100 mg/dL to 35 mg/dL (Fig. 5d). In contrast, the CAD risk sub-groups with non-high CAD-PRS achieved much more modest reductions in absolute and relative risk when going from an LDL of 100 mg/dL to 35 mg/dL (Fig. 5g).

For patients with HbA1c ≥ 6%, a diabetic or pre-diabetic threshold depending upon the national standard, the degree of absolute risk reduction achieved by HbA1c lowering increased with increasing T2D genetic risk (Fig. 5b). In this case there was little difference observed in the degree of risk reduction achieved at HbA1c targets of 6% (2.81% absolute risk reduction) vs a HbA1c target of 5.6% (2.89% absolute risk reduction). However, again we observed sub-groups achieving greater absolute and relative risk reduction levels through HbA1C lowering. Notably, two sub-groups (blue and purple) achieved similar and increased benefit from HbA1c lowering, with increasing benefit across HbA1c targets. These two sub-groups are enriched with diabetic individuals relative to other sub-groups except for the highest risk sub-groups.

For patients with SBP ≥ 140 mmHg, a similar trend of increasing absolute risk reduction with increasing SBP PRS is observed, though with the uncertainty in the SBP-PRS apparent in the risk trajectories, with high genetic risk individuals achieving 1.11% absolute risk reduction at an SBP target of 120 mmHg and 1.23% at an SBP target of 110 mmHg (Fig. 5c). Markedly different trajectories in risk reduction achieved by SBP lowering can be observed across the CAD risk subgroups (Fig. 5f,i). Notably, the highest CAD genetic risk sub-group (red) achieves the least benefit from SBP lowering, whereas the second highest CAD genetic risk sub-group (orange) achieves the greatest degree of absolute and relative risk reduction from SBP lowering. The optimal SBP target for this SBP responsive sub-group appears to be lower (100 mmHg) than the optimal SBP target for other sub-groups (110 mmHg). All sub-groups display increasing risk when SBP is overly lowered (≤ 90mmHg) and increasing risk above 140 mmHg (Fig. 5f,i).

## Discussion

Here we demonstrate that our meta-prediction framework, which appropriately integrates genetic and non-genetic risk factors into an ensemble prospective risk prediction model, produces superior and more generalizable risk predictions relative to the current clinical standard as well as existing linear / percentile-based genetic risk stratification approaches. Individualized risk reduction profiles are captured natively by this model and are most strongly linked to differences in genetic risk background across individuals, without any engineering of the model to produce such differential risk reduction profiles. This work serves as a clear demonstration of the importance and utility of incorporating genetic risk into modern risk stratification approaches for clinical prevention, both for risk detection and for clinical-decision support.

One important strength of our approach is the comprehensive dissection of CAD risk factors into past, present, and future measures and projections of risk, handling modifiable and unmodifiable risk factors in a manner that allows for the separate and combined characterization of inborn vs acquired sources of risk. The substantially greater importance of meta-features incorporating only unmodifiable sources of risk further underscores the importance of genetic risk in producing superior and actionable risk profiles. Genetic risk for CAD, CAD components, other late cardiovascular outcomes like cardiomyopathy, contributing diagnoses like T2D and CKD, biomarkers linked to CAD risk including lipid levels, waist-hip ratio, and coagulation factors, and other diagnoses like depression and lung cancer all contribute to the genetic landscape driving CAD risk and personalized avenues for intervention. While we only project the benefits achieved by modulating standard clinical risk factors (LDL, glucose, blood pressure), it is likely that the plethora of polygenic risk scores contributing to this prediction underlie further opportunities for personalized health interventions. Furthermore, our approach to transforming basic unmodifiable risk factors like age and sex into more complex, risk-factor specific, unmodifiable risk profiles highlight the importance of personalizing even the most basic of risk factors. While age and sex are the major components of PCE risk, leading to highly non-personalized projections of risk, age and sex are not used as standalone predictors in our model. This ultimately results in the detection of risk among those individuals typically considered low risk under current standards.

In fact, most patients diagnosed with premature myocardial infarction (< 55 years old) are not identified as at risk before their event by the guideline standard PCE 10-year ASCVD risk estimator^75^. There has also been concern regarding the significant overestimation of events by PCE and QRISK3^76,77^. Our model is able to overcome some of the flaws of these scores, particularly by identifying younger individuals who are at risk, identifying individuals at risk despite low PCE / QRISK, as well as identifying people not at risk despite elevated traditional risk factors. 20% of individuals without an event over the 10-year follow-up period were identified as at risk by PCE > 7.5% but not at risk by our meta-prediction model (Table 2). Similarly, > 10% of the population with an event over the 10-year follow-up period were identified as low risk by PCE / QRISK but captured as high risk by our model. These results suggest that our framework has the potential to overcome the key limitations of the current clinical standard.

A weakness of our work is the lack of independent replication. However, through careful train, validation, and test splits of the UK Biobank dataset, we were able to derive a robust model that recapitulates observations and clinical standards derived from external datasets.

For example, Mega et al. initially reported 1% – 8% absolute risk reduction by LDL lowering in high genetic risk individuals vs 0% – 2% absolute risk reduction by LDL lowering in low genetic risk individuals across several primary and secondary prevention trials^6^ – these values correspond to the absolute risk reduction profiles produced by our independent ML model. Current cholesterol control guidelines include intensive-cholesterol lowering (target < 70mg/dL) therapy for individuals at high CAD risk due to traditional CAD risk factors^78,79^. Our model supports this conclusion and suggests that genetic risk profiles can help identify at-risk individuals who may benefit from intensive cholesterol-lowering therapy, further supporting targets of < 35 mg/dL proposed by some clinical guidelines for high-risk individuals^80^. At the same time, our results support the conclusion that there is little benefit to reducing LDL below 70–100 mg/dL for individuals not at high risk, in line with current European guidelines (Fig. 5d)^79^.

The 2017 American BP guidelines state that a SBP < 130 mmHg may be a reasonable target for hypertensive adults without additional markers of increased cardiovascular risk while the 2023 European guidelines support SBP < 130 mmHg for hypertensive individuals^81,82^. In general, there is increasingly compelling evidence supporting the universal SBP goal of < 130 mmHg^83–86^. Our results support this, where model inference results in the majority of risk reduction being achieved at an SBP of 130 mmHg. However, our study also supports optimal SBP targets between 100 and 110 mmHg. A recent meta-analysis supports this finding by showing a strong and continuous dose-response between SBP levels and CAD risk from 100 to 200 mmHg, with optimal BP under 120 mmHg^87^. With excessive SBP lowering, we observe slightly increased risk of CAD, which has been previously observed in clinical studies (Fig. 5i)^88,89^.

Similarly, clinical trials targeting HbA1c to near-normal levels using intensive glucose-lowering therapy have failed to demonstrate any benefit despite the fact that T2D is a major established risk factor for CAD^90–92^. The small degree of absolute risk reduction observed via glucose lowering is consistent with our findings (Fig. 5e). A recent study showed that intensive glucose-lowering therapy was beneficial in preventing incident CAD only in a subset of diabetic patients defined by haptoglobin type, implying that genotypic and/or phenotypic differences can mediate the cardioprotective effects of intensive glucose-lowering therapy^93^. Our results support this possibility as the additional CAD risk reduction observed at the HbA1c level below 6%, the usual target value of intensive glucose-lowering therapy, was confined to two of the five subgroups classified according to genetic and phenotypic features (Fig. 5e).

Collectively, these findings suggest that meta-prediction framework can be used as a tool to plan and prioritize clinical intervention strategies and to tailor the therapeutic targets of each intervention to individual subjects, which may promote improved personalized care for primary CAD prevention. While our results are promising, we recognize the need for further research and external validation. Our findings must be tested across diverse populations and in various clinical settings to solidify the reliability and applicability of our framework. The pursuit of such confirmatory studies will be critical in refining our predictive tool and in ensuring that it can serve as a robust platform for the personalized prevention of CAD.

Improving the meta-prediction framework’s performance could involve several enhancements. Longitudinal biomarker and lifestyle data could add valuable temporal dimensions to risk profiling, capturing the evolving risk profile of an individual. Similarly, improvements in the capture of detailed EHR data would enable a more precise assessment of the duration of contributing diagnoses and a more accurate interpretation of those contributing risk factors. Furthermore, the integration of comprehensive ‘omics’ data—beyond genomics to include epigenomics, transcriptomics, proteomics, and metabolomics - could provide a means to measure the outcome of gene by environment interactions longitudinally, though multiple ‘omics assessments are likely not practical in real-world scenarios. In addition, widening the dataset to include a more diverse population, in terms of both ages and ancestry, could help to ensure that the model’s predictions are universally applicable. Although our study encompasses social determinants, enhancing the granularity and quality of this data could offer deeper insights into how these factors interact with biomarkers to influence individual health trajectories.

In summary, we present a fully integrative meta-prediction framework significantly outperforming current research and clinical standards for prospective prediction, especially in sub-groups of individuals traditionally considered low risk. This novel framework produces actionable predictions with magnitude of risk reduction inline with known guideline scenarios, and perhaps provides a basis for reconciling different risk factor targets across various national guidelines by identifying sub-groups differentially responsive to the more aggressive analyte thresholds proposed across those various guidelines. Finally, the genetic risk profile of individuals is the major factor driving the separation of these sub-groups, mediating differential benefit of standard interventions. Our work is a demonstration of the power of appropriately applying genetic risk early during risk stratification and in a manner that accounts for the heterogeneity of risk profiles apparent in a real-world population.

## Figures and Tables

**Table 1:** Baseline characteristics of the UK Biobank participants in the study (n = 339,390)

Variable	Count (%) or mean (± SD)	
	Prevalent CAD cohort (N = 172,296)	Incident CAD cohort (N = 167,094)
**Demographic indicator**		
Age (year)	57.76 (± 8.0)	57.21 (±8.0)
Sex (male)	92,703 (53.8%)	94,145 (56.34%)
**Diagnosis**		
Coronary artery disease	16,301 (9.46%)	0 (0.0%)
Myocardial infarction	11,860 (6.88%)	0 (0.0%)
Revascularization	10,269 (5.96%)	0 (0.0%)
Angina	17,271 (10.02%)	5,281 (3.16%)
Atherosclerotic cardiovascular disease	20,572 (11.94%)	5,031 (3.01%)
Abdominal aortic aneurysm	346 (0.2%)	152 (0.09%)
Peripheral artery disease	1,850 (1.07%)	870 (0.52%)
Non-stroke (pre-)cerebral disease	731 (0.42%)	237 (0.14%)
Type 1 diabetes	1,247 (0.72%)	960 (0.57%)
Type2 diabetes	5,990 (3.48%)	4,223 (2.53%)
Chronic kidney disease	582 (0.34%)	472 (0.28%)
**Lifestyle**		
Smoking status (previous/current)	64,695/19,667 (37.55%/11.41%)	59,702/18,261 (35.73%/10.93%)
Alcohol drinker (previous/current)	7,296/156,862 (4.23%/91.04%)	6,623/152,626 (3.96%/91.34%)
**Medication (self-reported or verbal interview)**		
Cholesterol lowering	48,183 (27.97%)	36,547 (21.87%)
Blood pressure	46,277 (26.86%)	37,574 (22.49%)
Insulin	2,652 (1.54%)	2,216 (1.33%)
Antiplatelet	35,992 (20.89%)	24,161 (14.46%)
Hormone replacement therapy	16,648 (9.66%)	16,124 (9.65%)
**Family history**		
Heart disease	83,647 (48.55%)	79,339 (47.48%)
Stroke	50,292 (29.19%)	48,767 (29.19%)
High blood pressure	87,445 (50.75%)	85,733 (51.31%)
Diabetes	41,053 (23.83%)	39,829 (23.84%)
**Body composition**		
BMI	27.78 (± 4.96)	27.70 (± 4.9)
Waist-hip ratio	0.88 (±0.09)	0.87 (±0.09)
**Cardiovascular metric**		
Systolic blood pressure (mmHg)	138.14 (±18.76)	138.41 (±18.72)
Diastolic blood pressure (mmHg)	81.98 (±10.21)	82.47 (±10.13)
**Biomarker**		
Albumin (g/L)	45.02 (± 2.65)	45.09 (± 2.62)
Cholesterol (mmol/L)	5.58 (±1.19)	5.71 (±1.15)
Creatinine (umol/L)	72.88 (± 20.12)	72.01 (±19.86)
C-reactive protein (mg/L)	2.85 (± 4.72)	2.78 (± 4.54)
Cystatin C (mg/L)	0.93 (± 0.2)	0.91 (±0.18)
Glucose (mmol/L)	5.18 (±1.34)	5.14 (±1.27)
Glycated hemoglobin (HbA1c) (mmol/mol)	36.69 (± 7.35)	36.36 (± 6.92)
HDL cholesterol (mmol/L)	1.43 (±0.39)	1.45 (±0.38)
LDL cholesterol (mmolL)	3.48 (± 0.9)	3.57 (±0.87)
Total bilirubin (umol/L)	9.1 (±4.46)	9.01 (±4.38)
Triglycerides (mmol/L)	1.78 (± 1.04)	1.78 (± 1.04)
White blood cell leukocyte count (10^9^ cells/L)	7.01 (± 2.21)	6.95 (± 2.12)
Hemoglobin concentration (g/dL)	14.14 (± 1.27)	14.14 (± 1.26)
Cholesterol HDL ratio	4.10 (± 1.13)	4.15 (± 1.14)
**Clinical risk score**		
PCE	9.04 (± 7.72)	8.48 (± 7.36)
QRISK3	15.81 (±11.42)	14.85 (±10.66)

**Table 2:** Reclassification table

	No event (n = 30,257)		Events(n = 3, 162)		Net reclassification index (NRI)
Meta-prediction	Negative (< 0.24)	Positive (≥ 0.24)	Negative (< 0.24)	Positive (≥ 0.24)	
PCE					(19.57% + 1.72%) – (2.86% + 3.16%) + (4.97% + 6.33%) – (11.80% + 2.02) = 0.13
Low (< 5%)	41.47%	2.86%	8.76%	4.97%	
Borderline (5–7.5%)	11.68%	3.16%	4.71%	6.33%	
Intermediate (7.5–20%)	19.57%	14.68%	11.80%	40.73%	
High (≥ 20%)	1.72%	4.85%	2.02%	20.68%	
QRISK3					(26.03% + 8.94%) – (2.41%) + (4.02%) – (11.86% + 7.62%) = 0.17
Low (< 10%)	39.47%	2.41%	7.81%	4.02%	
Moderate (10–20%)	26.03%	9.22%	11.86%	21.06%	
High (≥ 20%)	8.94%	13.93%	7.62%	47.63%	
GPS_CAD_					(15.16%) – (8.21%) + (15.28%) – (11.01%) = 0.11
Negative (< 0.09)	59.28%	8..21%	16.29%	15.28%	
Positive (≥ 0.09)	15.16%	17.35%	11.01%	57.43%	
metaGRS_CAD_					(16.75%) – (7.50%) + (14.04%) – (11.80%) = 0.11
Negative (0.09)	57.67%	7.50%	15.50%	14.04%	
Positive (≥ 0.09)	16.78%	18.05%	11.80%	58.67%	

Percentage of participants in test set of incident CAD cohort (n = 33,419) classified in each risk tier of PCE and QRISK3, as well as GPS_CAD_ and metaGRS_CAD_ vs. meta-prediction at the optimal risk threshold by maximizing Youden’s index.

**Table 3:** Characteristics of the risk sub-groups in the study

Variable	Count (%) or mean (± SD)				
	highest risk sub-group (N = 11,772)	higher risk sub-group (N = 33,028)	medium risk sub-group (N = 21,511)	lower risk sub-group (N = 15,082)	lowest risk sub-group (N = 50,224)
**Demographic indicator**					
Age (year)	61.31 (± 6.09)	59.69 (± 6.83)	55.99 (± 8.18)	58.12 (± 7.4)	55.64 (± 8.45)
Sex (male)	10,150 (86.22%)	18,993 (57.51%)	9,566 (44.47%)	5,948 (39.44%)	16,845 (33.54%)
**Diagnosis**					
Angina	944 (8.02%)	1,128 (3.42%)	782 (3.64%)	608 (4.03%)	1,446 (2.88%)
Atherosclerotic cardiovascular disease	835 (7.09%)	1,046 (3.17%)	827 (3.84%)	575 (3.81%)	1,317 (2.62%)
Abdominal aortic aneurysm	28 (0.24%)	48 (0.15%)	26 (0.12%)	13 (0.09%)	29 (0.06%)
Peripheral artery disease	121 (1.03%)	197 (0.6%)	120 (0.56%)	78 (0.52%)	250 (0.5%)
Non-stroke (pre-) cerebral disease	54 (0.46%)	49 (0.15%)	38 (0.18%)	25 (0.17%)	50 (0.1%)
Type 1 diabetes	56 (0.48%)	143 (0.43%)	23 (0.11%)	207 (1.37%)	500 (1.0%)
Type 2 diabetes	538 (4.57%)	736 (2.23%)	353 (1.64%)	706 (4.68%)	1,665 (3.32%)
Chronic kidney disease	38 (0.32%)	94 (0.28%)	116 (0.54%)	66 (0.44%)	108 (0.22%)
**Lifestyle**					
Smoking status (previous/current)	5,168/1,234 (43.9%/10.48%)	12,956/2,174 (39.23%/6.58%)	6,549/5,568 (30.44%/25.88%)	5,777/1,904 (38.3%/12.62%)	16,524/4,850 (32.9%/9.66%)
Alcohol drinker (previous/current)	511/10,812 (4.34%/91.85%)	1,224/30,415 (3.71%/92.09%)	1,043/19,422 (4.85%/90.29%)	734/13,593 (4.87%/90.13%)	1876/45,700 (3.74%/90.99%)
**Medication (self-reported or verbal interview)**					
Cholesterol lowering	3,989 (33.89%)	6,350 (19.23%)	5,495 (25.55%)	3,342 (22.16%)	6,922 (13.78%)
Blood pressure	4,410 (37.46%)	8,259 (25.01%)	5,507 (25.6%)	3,831 (25.4%)	9,966 (19.84%)
Insulin	182 (1.55%)	329 (1.0%)	33 (0.15%)	486 (3.22%)	1,124 (2.24%)
Antiplatelet	3,063 (26.02%)	4,925 (14.91%)	4,857 (22.58%)	2,554 (16.93%)	5,709 (11.37%)
Hormone replacement therapy	1,132 (9.62%)	3,378 (10.23%)	2,137 (9.93%)	1,377 (9.13%)	4,621 (9.2%)
**Family history**					
Heart disease	8,436 (71.66%)	19,020 (57.59%)	9,249 (43.0%)	7,812 (51.8%)	18,733 (37.3%)
Stroke	3,714 (31.55%)	10,376 (31.42%)	6,301 (29.29%)	4,575 (30.33%)	13,796 (27.47%)
High blood pressure	5,847 (49.67%)	16,719 (50.62%)	11,109 (51.64%)	7,751 (51.39%)	26,020 (51.81%)
Diabetes	2,841 (24.13%)	7,707 (23.33%)	5,217 (24.25%)	3,835 (25.43%)	12,286 (24.46%)
**Body composition**					
BMI	28.34 (± 4.44)	27.53 (± 4.41)	28.0 (± 5.24)	28.48 (± 5.11)	27.78 (± 5.19)
Waist-hip ratio	0.93 (± 0.08)	0.89 (± 0.09)	0.88 (± 0.08)	0.88 (± 0.09)	0.86 (± 0.09)
**Cardiovascular metric**					
Systolic blood pressure (mmHg)	145.63 (± 18.24)	142.39 (± 18.23)	135.5 (± 16.1)	137.98 (± 18.81)	137.77 (± 20.08)
Diastolic blood pressure (mmHg)	84.69 (± 10.1)	83.6 (± 9.9)	81.95 (± 9.67)	82.19 (± 10.19)	82.32 (± 10.54)
**Biomarker**					
Albumin (g/L)	45.14 (± 2.61)	45.14 (± 2.57)	45.01 (± 2.71)	44.9 (± 2.62)	45.12 (± 2.64)
Cholesterol (mmol/L)	5.52 (± 1.18)	5.78 (± 1.16)	5.55 (± 1.15)	5.74 (± 1.2)	5.76 (± 1.16)
Creatinine (umol/L)	79.72 (± 17.33)	74.24 (± 19.53)	73.29 (± 24.01)	72.03 (± 22.93)	69.9 (± 19.53)
C-reactive protein (mg/L)	2.96 (± 4.93)	2.58 (± 4.33)	3.23 (± 5.17)	3.04 (± 4.67)	2.82 (± 4.57)
Cystatin C (mg/L)	0.97 (± 0.18)	0.92 (± 0.17)	0.95 (± 0.2)	0.94 (± 0.21)	0.9 (± 0.17)
Glucose (mmol/L)	5.3 (± 1.44)	5.13 (± 1.13)	5.01 (± 0.81)	5.36 (± 1.79)	5.22 (±1.51)
Glycated hemoglobin (HbA1c) (mmol/mol)	37.74 (± 7.87)	36.23 (± 6.29)	35.95 (± 4.64)	37.94 (± 9.41)	36.71 (± 8.15)
HDL cholesterol (mmol/L)	1.28 (± 0.33)	1.43 (± 0.39)	1.38 (± 0.35)	1.39 (± 0.37)	1.49 (± 0.39)
LDL cholesterol (mmolL)	3.5 (± 0.89)	3.63 (± 0.88)	3.48 (± 0.88)	3.62 (± 0.91)	3.58 (± 0.88)
Total bilirubin (umol/L)	9.88 (± 4.72)	9.36 (± 4.5)	8.77 (± 4.41)	8.78 (± 4.17)	8.81 (± 4.23)
Triglycerides (mmol/L)	2.07 (± 1.18)	1.83 (± 1.05)	1.84 (± 1.03)	1.92 (± 1.09)	1.72 (± 1.04)
White blood cell leukocyte count (10^9^ cells/L)	7.14 (± 2.06)	6.81 (± 1.99)	7.47 (± 2.07)	6.96 (± 1.88)	6.95 (± 2.32)
Hemoglobin concentration (g/dL)	14.78 (± 1.16)	14.36 (± 1.23)	14.23 (± 1.25)	14.09 (± 1.26)	13.97 (± 1.26)
Cholesterol HDL ratio	4.49 (± 1.17)	4.25 (± 1.16)	4.21 (± 1.14)	4.34 (± 1.15)	4.06 (± 1.15)
**Clinical risk score**					
PCE	15.12 (± 8.14)	10.66 (± 7.64)	7.94 (± 6.46)	8.81 (± 6.91)	7.23 (± 7.26)
QRISK3	24.44 (± 11.03)	17.93 (± 10.56)	14.02 (± 9.28)	16.25 (± 10.77)	12.91 (± 10.87)
**Polygenic risk score**					
CAD-PRS (PGS003356)	1.3152 (± 0.7885)	0.4992 (± 0.8523)	−0.0867 (± 0.8616)	−0.1145 (± 0.8823)	−0.3585 (± 0.9127)
T2D-PRS (PGS000330)	0.2049 (± 0.8777)	0.0214 (± 0.9132)	−0.0101 (± 0.993)	0.0345 (± 0.9779)	0.0162 (± 1.0842)
SBP-PRS (PGS000301)	0.2447 (± 0.9854)	0.1007 (± 0.992)	−0.0234 (± 0.9994)	−0.0318 (± 0.9971)	−0.0633 (± 1.0001)
TGs-PRS (PGS002287)	0.1108 (± 0.9105)	0.0442 (± 0.9571)	0.0118 (± 1.0029)	0.0145 (± 0.9952)	−0.0446 (± 1.0482)
HbA1c-PRS (PGS001352)	0.0949 (± 1.0029)	0.0255 (± 0.9897)	0.001 (± 1.001)	0.0041 (± 0.9872)	−0.0181 (± 0.9972)
WHR-PRS (PGS000299)	0.098 (± 0.9968)	0.0307 (± 1.0067)	0.026 (± 1.0068)	0.0067 (± 0.998)	−0.0256 (± 0.9974)
BMI-PRS (PGS000910)	0.2033 (± 0.9676)	0.0561 (± 0.9707)	0.048 (± 1.0109)	0.0861 (± 0.9936)	0.0162 (± 1.0208)
**Meta-prediction**	0.75 (± 0.27)	0.33 (± 0.32)	0.18 (± 0.25)	0.17 (± 0.23)	0.15 (± 0.25)

## Data Availability

All data are made available from the UK Biobank (https://www.ukbiobank.ac.uk/enable-your-research/apply-for-access) to researchers from universities and other institutions with genuine research inquiries following institutional review board and UK Biobank approval. This research has been conducted using the UK Biobank Resource under Application Number 41999.
